# In Situ Synthesis of a Hydroxyapatite and Reduced Graphene Oxide Composite for Potential Electrochemical Biosensing Applications

**DOI:** 10.1021/acsomega.5c03514

**Published:** 2025-07-04

**Authors:** José J. Ruíz-Osorio, R. Aguilar-Sánchez, Rutilo Silva-González, Ana K. Sánchez-Hernández, Mohammad N. Banis, Jian Wang, M. J. Robles-Águila

**Affiliations:** † Centro de Investigación en Dispositivos Semiconductores, Benemérita Universidad Autónoma de Puebla, Instituto de Ciencias, Edificio IC 6, Boulevard 14 Sur y Av. San Claudio, Col San Manuel, Puebla C. P. 72570, México; ‡ Facultad de Ciencias Químicas, Benemérita Universidad Autónoma de Puebla., Puebla 72420, México; § Instituto de Física, Benemérita Universidad Autónoma de Puebla, Apartado Postal J-48, Puebla 72570, México; ∥ Department of Mechanical and Materials Engineering, University of Western Ontario, London N6A 3K7, Canada; ⊥ 117197 Canadian Light Source Inc, Saskatoon, Saskatchewan S7N 2 V3, Canada

## Abstract

Hydroxyapatite/reduced graphene oxide (HA/rGO) composites are extensively used in numerous applications, including tissue engineering, energy storage, catalysis, and electrochemical sensing. In this study, a novel microwave hydrothermal-assisted coprecipitation method was implemented to synthesize an in situ HA/rGO composite for potential electrochemical biosensing applications. The structural, optical, and morphological properties were thoroughly analyzed using XRD, Raman spectroscopy, FTIR, STXM, and SEM. Rietveld refinement confirmed the presence of a hexagonal crystalline phase in the HA/rGO composite with a mean crystallite size of 28.1 nm. Raman spectroscopy revealed characteristic vibrational modes of each precursor, while STXM spectra displayed electronic transitions corresponding to rGO (C 1s to π* and σ* levels) as well as Ca L-edge and O and P K-edge transitions of HA, confirming a composite material. FTIR analysis confirmed the reduction of GO to rGO by tracking the presence and disappearance of oxygen-functional groups in the graphitic structure. Electron microscopy revealed that HA nanorods, averaging 75 nm in length, were uniformly distributed along the surface and edges of the rGO layers.

## Graphical abstract

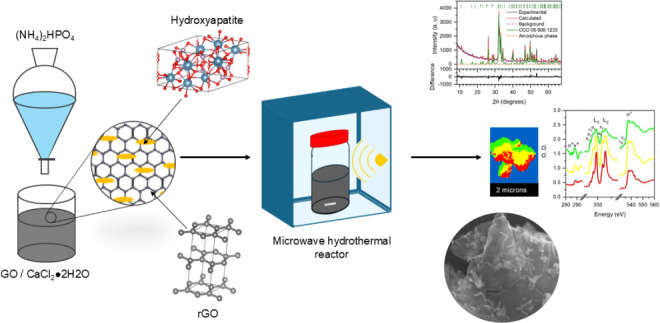

## 1 Introduction

Hydroxyapatite (HA) is a bioceramic material widely recognized for its notable bioactivity, biocompatibility, and structural similarity to the mineral phase of bone tissue, which makes it highly suitable for biomedical applications, particularly in bone regeneration and implant coatings.^1,2^ However, the low mechanical strength of HA presents limitations for broader applications, especially in electrochemical biosensing, where stability and conductivity are crucial factors to achieve effective results.^3,4^ Graphene-based materials, particularly reduced graphene oxide (rGO), have attracted considerable attention due to their excellent electrical conductivity, high surface area, and mechanical robustness.^5–9^ The incorporation of rGO into HA has emerged as a promising strategy to enhance the mechanical, structural, and electrochemical properties of HA while preserving its biocompatibility.^10,11^ Several research studies have focused on this composite material due to its potential applications in energy storage devices, catalysis, and electrochemical sensing.^12,13^ The incorporation of rGO improves the electron transfer properties of HA, which, in turn, enhances the sensing capabilities for biomedical applications, such as enzymatic and nonenzymatic biosensors.^10,14,15^ Numerous synthesis routes have been explored to fabricate HA/rGO composites, including the sol–gel, sonochemistry, hydrothermal, microwave, and coprecipitation methods. However, the microwave-assisted hydrothermal method is a novel, promising, and effective route to obtain nanoparticles. This method achieves temperatures of 200 °C and 30 bar pressure, accelerating nucleation and crystal growth while significantly reducing the reaction time and energy consumption compared to traditional hydrothermal processes. Several reports indicated that elevated temperature and pressure enhance precursor solubility and dispersion, resulting in highly crystalline material, while the rapid heating rates provided by microwave irradiation generate smaller, uniformly shaped nanoparticles.^16–19^ Despite these advances, a fundamental understanding of the structural, morphological, and electrochemical interactions between HA and rGO remains an active area of research that needs further investigation. This study utilizes the microwave hydrothermal-assisted coprecipitation method to produce in situ synthesis of HA/rGO composites. The characterization of these materials provides insights into their phase composition, chemical interactions, and microstructural attributes, which highlight their potential use for electrochemical biosensing applications.

## 2 Experimental Section

### 2.1 Chemicals and Materials

All chemical reagents used in these experiments were purchased from commercial sources as analytical grade reagents and used directly without further purification.

The HA and the HA/rGO composite were synthesized by a novel microwave hydrothermal-assisted coprecipitation method following green chemistry principles. Calcium chloride dihydrate (CaCl_2_·2H_2_O) at 99% purity, ammonium phosphate dibasic ((NH_4_)_2_HPO_4_) at 98% purity, Trizma base (C_4_H_11_NO_3_) at 99.9% purity (brand Sigma-Aldrich), and ammonium hydroxide (NH_4_OH) at 28% purity (brand J. T. Baker) were used as reagents for the synthesis of HA.

The synthesis of rGO was carried out by the thermal reduction of GO, previously obtained by the Hummers’ method. Graphite powder (particle size <20 μm), sodium nitrate (NaNO_3_) at >99% purity (brand Sigma-Aldrich), sulfuric acid (H_2_SO_4_) at >95% purity (brand Meyer), potassium permanganate (KMnO_4_) at 99% purity (brand Karal), and hydrogen peroxide (H_2_O_2_) at 30.3% purity (brand Fermont) were used as reagents.

### 2.2 Synthesis of Graphene Oxide (GO) and Reduced Graphene Oxide (rGO)

Graphene oxide (GO) was synthesized using Hummers’ method, as previously reported in the literature.^13,20^ Briefly, 23 mL of concentrated H_2_SO_4_ were placed in a beaker, followed by the addition of 1 g of graphite powder and 0.5 g of NaNO_3_. The mixture was stirred vigorously while maintaining the reaction temperature below 10 °C for 30 min. Subsequently, 3 g of KMnO_4_ was gradually added over a period of 2 h. The temperature was increased to 35 °C and maintained for 30 min. Afterward, 46 mL of hot deionized water (DI H_2_O) was added, and the reaction was stirred for an additional 15 min at 98 °C, leading to a dark brown reaction coloration. Next, 140 mL of DI H_2_O and 3 mL of H_2_O_2_ were added while maintaining the temperature at 98 °C. The resulting black, opaque solution was washed with DI H_2_O and 5% HCl by centrifugation at 19,000 rpm for 35 min. Finally, the obtained precipitate was dried in a hot air oven (Prendo model HSCF-30) at 70 °C for 24 h.

To synthesize reduced graphene oxide (rGO), the appropriate amount of graphene oxide (GO) was dispersed in deionized (DI) water and subjected to ultrasonication in a Cole–Parmer ultrasonic bath for 1 h to promote initial exfoliation. The resulting suspension was then further sonicated using a microtip probe in an ultrasonic processor (Sonics and Materials, Inc., Newton, CT, USA), operating at a frequency of 20 kHz and a power of 750 W with a 1 s pulse interval, for 30 min. Following sonication, the exfoliated GO suspension was centrifuged at 19,000 rpm for 35 min to remove unexfoliated material and impurities. Subsequently, the recovered GO sample was thermally reduced in a muffle furnace (Prendo model M1) at 350 °C for 4 h to eliminate oxygen-containing functional groups from the graphitic layer. Finally, the resulting rGO powder was ground using an agate mortar and then stored for further use.

### 2.3 Synthesis of Hydroxyapatite (HA)

The HA was synthesized by using a wet chemical approach via the microwave-assisted hydrothermal method. In a typical synthesis, a 0.2 M solution of (NH_4_)_2_HPO_4_ was added dropwise to a 0.24 M CaCl_2_·2H_2_O solution. Both calcium and phosphate precursor solutions were prepared by using a 0.1 M Trizma base solution. The pH value of the solutions was set to 10.5 using NH_4_OH. The HA precursor solution was stirred magnetically for 30 min until the formation of a homogeneous sol was observed. Subsequently, the mixture was transferred to a Monowave 400 Microwave Synthesis Reactor (Anton Paar) for microwave-assisted hydrothermal treatment. The conditions of the reaction were set at a 200 °C temperature, *a* < 25-bar pressure, and a 15 min time. Subsequently, the resulting solution was washed several times with deionized (DI) water and ethanol. The gel was aged at room temperature for 24 h. After that, the sample was dried in a hot air oven at 100 °C for 12 h and 200 °C for 1 h to eliminate adsorbed water in the sample and to favor the formation of xerogel. Finally, the powder was grounded in an agate mortar and stored for further use.

### 2.4 Synthesis of Hydroxyapatite and Reduced Graphene Oxide (HA/rGO) Composite

The HA/rGO composite was synthesized via the in situ growth of hydroxyapatite (HA) on reduced graphene oxide (rGO) sheets using a microwave hydrothermal-assisted coprecipitation method. In this process, 100 mg of previously synthesized GO was dispersed in a Trizma base solution and sonicated in an ultrasonic bath for 30 min. Subsequently, the appropriate amount of CaCl_2_·2H_2_O was added to the GO suspension, which was then maintained under sonication for an additional 30 min. In parallel, a 0.2 M (NH_4_)_2_HPO_4_ solution was prepared by using the same Trizma base. The pH values of both solutions were adjusted to a pH value >10.5 with a NH_4_OH solution. Subsequently, the (NH_4_)_2_HPO_4_ solution was added to the GO/CaCl_2_ suspension dropwise to favor the interaction between the Ca^2+^ and PO_4_^3–^ ion precursor solution and form the HA crystal on the GO sheet. The following stages of the synthesis procedure were the same as those carried out for the HA synthesis, until a HA/rGO composite powder was finally obtained. The samples were labeled as HA and HR for hydroxyapatite and HA/rGO composite, respectively. A schematic diagram of the synthesis of each sample is illustrated in Scheme 1.

**Scheme 1:**
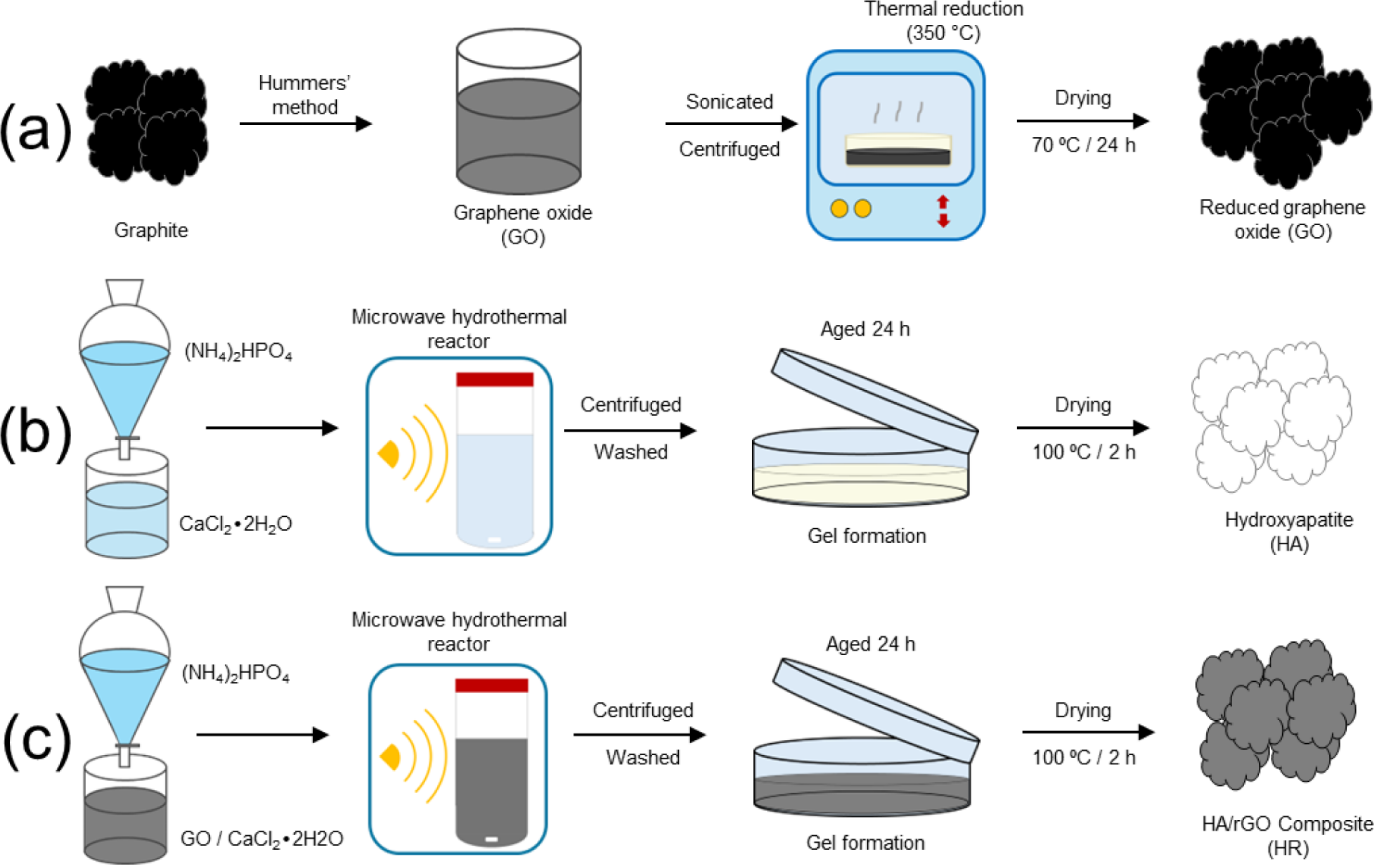
Schematic Diagram Illustrating the Synthesis of the (a) rGO, (b) HA, and (c) HA/rGO Composite

### 2.5 Characterization of the Materials

The X-ray powder diffraction patterns of the GO, rGO, HA, and HA/rGO composites were obtained with an Empyrean Panalytical diffractometer with a CuKα1 radiation of 1.5406 Å. The data were collected in the 2θ range from 7.5 to 70° with a step size of 0.02°. The QualX2 software version 2.24 and the PowCod 2205 database^21^ from the Institute of Crystallography-CNR Bari were used to investigate the crystalline phase of each sample. In addition, Rietveld refinement was performed using the BGMN/Autoquan software version 4.2.22 ^22^ with the graphical user interface Profex version 5.2.3.^23^ The incoming crystallographic data for the Rietveld refinement were taken from the Crystallography Open Database (COD) file 00-900-1233 ^24^ and fixed for all the refinements. The profile background was modeled with a 9-coefficient polynomial approach. The anisotropic crystallite size and isotropic microstrains were considered for line broadening. The refinement analysis was accomplished until a close fit between the experimental and calculated patterns was observed. The crystal structure (CIF file) of phases was plotted with VESTA software.^25^

Micro-Raman spectroscopy was carried out with a LabRAM HR-Olympus Micro-Raman System (HORIBA Jobin Yvon Inc., Edison, NJ, USA) using a He–Ne laser with an excitation wavelength of 632.8 nm. Soft X-ray spectromicroscopy was measured with the STXM at the 10ID-1 spectromicroscopy (SM) beamline at the Canadian Light Source (CLS, Saskatoon, SK, Canada). The beamline, hosted on a 2.9 GeV electron storage ring, consisted of an APPLE II-type Elliptically Polarizing Undulator (EPU), a Plane Grating Monochromator (PGM) optimized for photons in the range from 130 to 2700 eV, and other beamline optics and components. The STXM analysis provided chemical speciation and quantitative analysis at a spatial resolution of ∼25 nm. The images were obtained by a mechanical raster scan of the sample. Similarly, the X-ray focusing device zone plate (ZP) was placed as a function of photon energy to maintain the focus on the sample, with the order sorting aperture (OSA) remaining stationary. Further details from the SM beamline, STXM experiments, and data analysis can be found at https://sm.lightsource.ca/about/beamline/. The STXM data processing was carried out with aXis2000 software (2019 version).^26^

Fourier transform infrared spectroscopy was carried out to identify the functional groups of the samples. The FTIR spectra were obtained in a transmittance mode using a Nicolet 6700 (Thermo Scientific) in the range from 4000 to 400 cm^–1^ with the potassium bromide (KBr) pellet method. Each sample was mixed with KBr in a ratio of 0.5:100 and pressed at 2 ton/cm^2^ for 5 min after being ground homogeneously with an agate mortar. A surface morphology analysis was carried out by a Field Emission Scanning Electron Microscopy (FESEM) measurement with an electron microscope JEOL JSM-7800 F.

## 3 Results and Discussion

### 3.1 X-Ray Diffraction

XRD studies were conducted to investigate the structural properties of the synthesized powdered samples. Figure 1a shows the X-ray diffraction plot for graphite, GO obtained by the Hummers’ method, and thermally rGO. The pattern of the graphite showed a sharp characteristic peak at 26.59° corresponding to a *d-*spacing value of 3.35 Å and associated with the (002) plane. Furthermore, other low-intensity peaks were found at 42.64°, 44.70°, and 54.70° over 2θ corresponding to the (002), (100), (001), and (004) diffraction planes, respectively, according to the COD file 00-900-8569. Following chemical oxidation via Hummers’ method, the GO pattern exhibited a sharp peak at 11.95° corresponding to the (001) plane and a low intense peak at 42.49° related to the (100) plane. The interlayer spacing (*d*-spacing) value corresponding to the intense diffraction peak was estimated at 7.39 Å, confirming the oxidation of graphite. This increase in spacing is attributed to the introduction of oxygen-containing functional groups, such as carbonyl, carboxyl, hydroxyl, and epoxy, between the layers and at the edges of the graphitic sheets through chemical oxidation.^27,28^ Due to thermal reduction, the XRD displayed a diffraction peak at 26.58° for the rGO, which was associated with the (002) plane and a *d-spacing* value of 3.39 Å, confirming that the GO was highly reduced.^28,29^ However, a noticeable broad peak was present at ∼24.5°, which, according to refs. 8 and 30, may imply a poor arrangement of graphene layers along the stacking direction (*c-axis*). The *d-spacing* value of the most intense peak was calculated using Bragg’s equation:

nλ=2dsinθ

where *n* is the integer, λ is the wavelength of the X-ray radiation (1.5406 Å), and θ is the angle between the incident and reflected rays.

**Figure 1:**
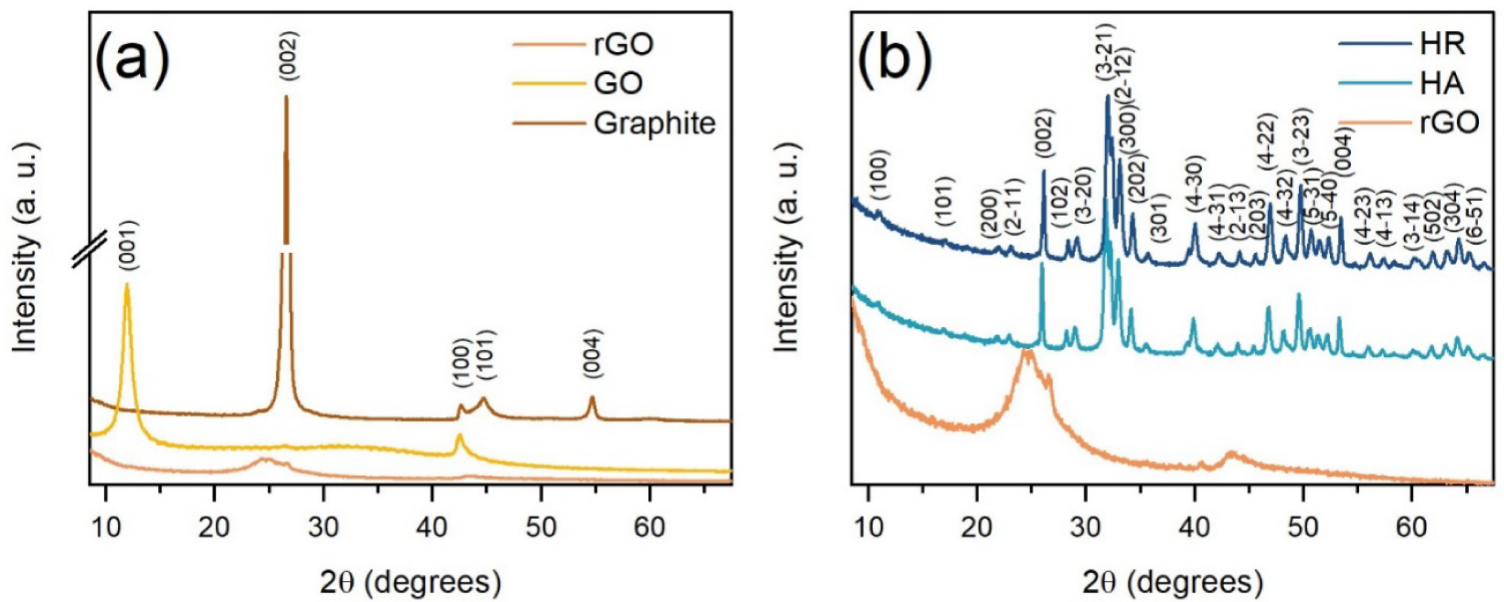
X-ray diffraction patterns of (a) the evolution of graphite to rGO by the Hummers’ method and US/thermal reduction and (b) XRD patterns of the HA/rGO composite and its precursors.

Figure 1b presents the XRD pattern for the rGO, HA, and HA/rGO composite. The HA pattern displays diffraction peaks associated with a hexagonal lattice of the HA crystal within the space group P6_3/m_, according to the COD file 00-900-1233. Notably, no additional peaks related to impurities or secondary phases are observed, indicating the high purity of the synthesized hydroxyapatite.^31,32^ Additionally, in the XRD pattern, the HA/rGO composite exhibits sharp, intense, and well-defined diffraction peaks corresponding to the hexagonal phase of HA, indicating that the crystalline structure of HA is preserved in the composite. The characteristic (002) diffraction peak of rGO is not observed, likely due to its significantly lower intensity and broader nature compared to the HA (002) peak, which can be attributed to the amorphous structure of rGO. Therefore, the rGO peak is covered by a highly intensified HA (002) peak with high crystallinity;^33^ however, a shift toward high angle values and a change in FWHM of the diffraction peak are estimated in the HR sample over the HA, which suggests the integration of each precursor during the microwave hydrothermal synthesis of the HA/rGO composite. Aiming a study of the influence of rGO on the HA nanostructure, lattice strain and dislocation density were estimated using the Williamson–Hall (W–H) method^34^ and the simplified Williamson–Smallman (W–S) approximation,^35^ respectively; the results can be seen in the Supporting Information.

Furthermore, Rietveld refinement was performed on the XRD data, and R_wp_ values of 6.99 and 8.33 were obtained for the HA and HA/rGO composite, respectively. A good fit was accomplished from the measured to the calculated XRD patterns. The hexagonal crystalline phase of the HA in both samples was confirmed. The cell parameters and crystallite size were obtained by this analysis and are summarized in Table 1. Figure 2 shows the Rietveld refinement plot for the HA and HR samples (see Supporting Information for Rietveld data).

**Figure 2:**
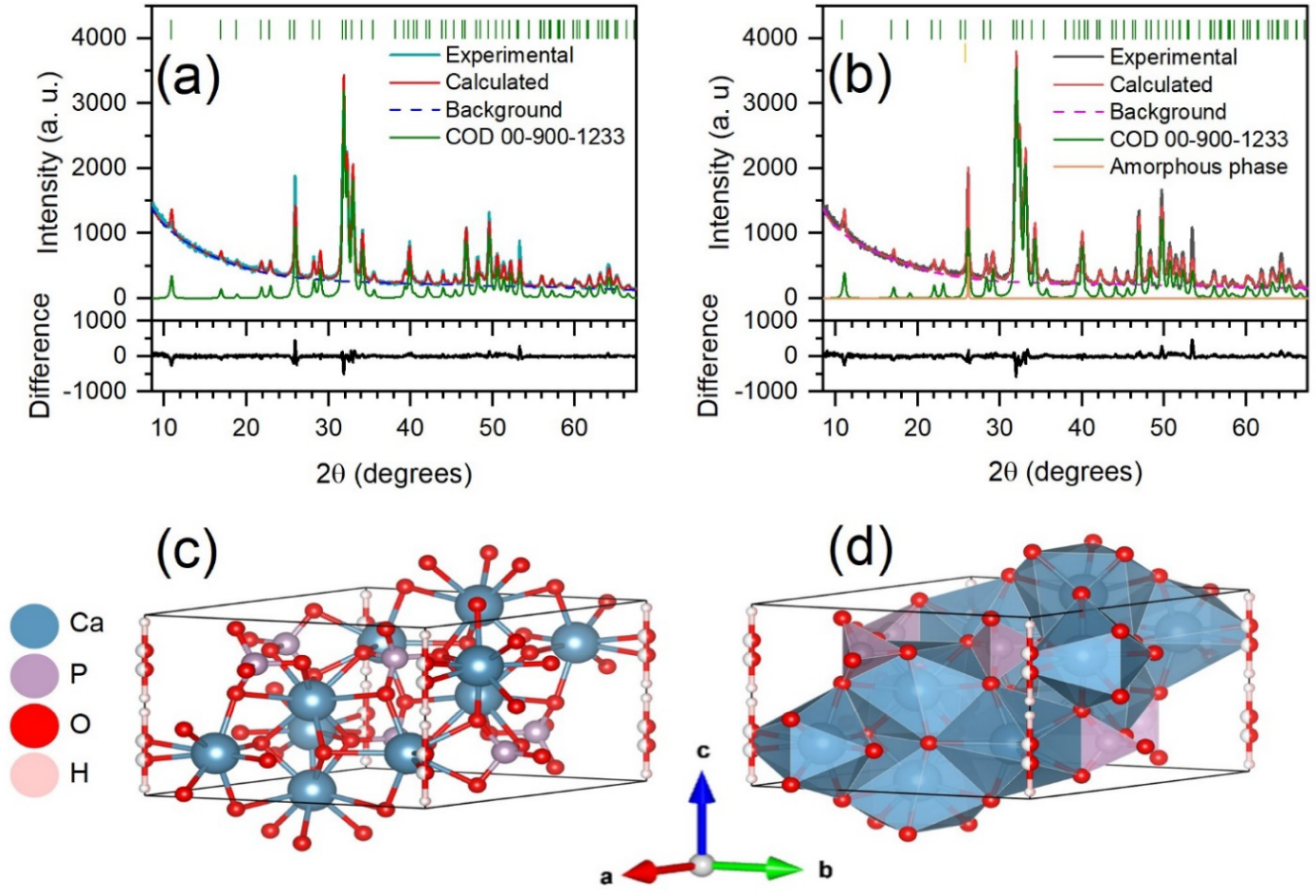
Rietveld refinement plots of (a) the HA and (b) the HA/rGO composite and (c) ball-and-stick and (d) polyhedral models of the refined structure along with the standard orientation of the crystal shape.

**Table 1:** Results of Rietveld Refinement Analysis

	Cell parameters					
Sample	*a* (Å)	*c* (Å)	Volume (Å^3^)	Crystallite size <100> (nm)	*R*_wp_	GoF (*R*_wp_/*R*_exp_)
HA	9.435 ± 0.006	6.886 ± 0.004	530.88	31.1±0.2	6.99	1.63
HR	9.451 ± 0.007	6.899 ± 0.005	533.72	28.5±0.2	8.33	2.01

### 3.2 Raman Spectroscopy

Raman spectroscopy is a high-quality, nondestructive technique used to investigate the structural properties of carbon-based materials, ceramics, metallic oxides, and composite materials. This technique confirmed the formation of the HA/rGO composite (Figure 3).

**Figure 3:**
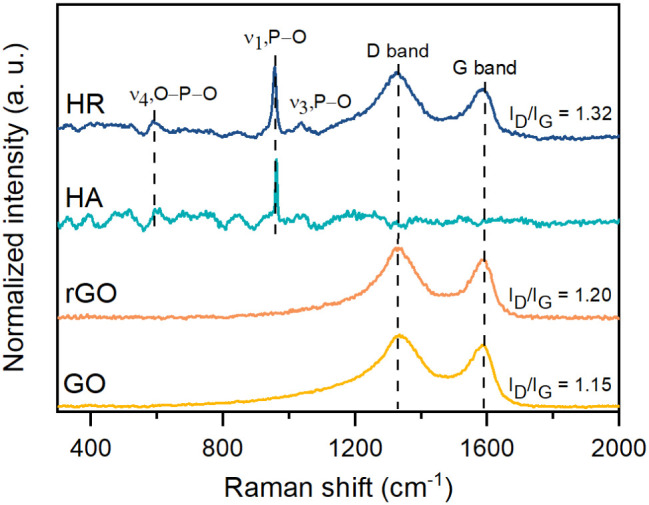
Raman spectra of the GO, rGO, HA, and HR samples.

Raman spectroscopy is a widely used technique to characterize the structural and electronic properties of graphene and its derivative compounds (graphite, GO, and rGO), including defects, density, and doping levels.^36^ In the graphene, GO, and rGO spectra, two characteristic bands are located in the range from 1200 to 1700 cm^–1^ associated with two fundamental vibrations of the crystal lattice, D and G bands.^29^ The D band, typically observed at 1355 cm^–1^, arises from the breathing mode of κ-point photons with A_1g_ symmetry and is associated with structural defects in the carbon lattice. Similarly, the G band, appearing at 1575 cm^–1^, originates from the first-order scattering of E_2g_ phonons by *sp^2^*-bonded carbon atoms, reflecting the graphitic nature of the material.^8,27,36^ Figure 3 shows that the D vibration bands of GO and rGO are recorded at 1333 and 1327 cm^–1^, respectively. Meanwhile, the G band is observed at 1583 and 1584 cm^–1^, respectively. The intensity ratio of the D to G band (*I*_D_/*I*_G_) is commonly used to assess the degree of graphitization and structural disorder in carbon-based materials. In this study, rGO exhibited a higher *I*_D_/*I*_G_ ratio (1.20) compared with GO (1.15), indicating an increase in structural defects. Additionally, the distance between defects (*L*_D_) and defect density (*n*_D_) values were calculated from the *I*_D_/*I*_G_ ratio using the equations proposed by Cançado et al.,^37^ to investigate the effect of chemical oxidation and thermal reduction processes in the graphitic structure. Relevant Raman parameters for GO and rGO are collected in Table 2.

**Table 2:** Raman Band Positions, *I*_D_/*I*_G_, *L*_D_, and *n*_D_ Values of GO and rGO

Sample	D-band (cm^–1^)	G-band (cm^–1^)	*I*_D_/*I*_G_ ratio	*L*_D_ (nm)	*n*_D_ × 10^–11^ (cm^–2^)
GO	1333	1583	1.15	15.8	1.29
rGO	1327	1584	1.20	15.5	1.35

Table 2 shows a correlation between the *I*_G_/*I*_D_ ratio and *L*_D_ in both GO and rGO samples. As is known, the intensity of the *I*_G_/*I*_D_ ratio is a metric that indicates the degree of defects introduced during the oxidation/reduction process from graphite to rGO. In that way, a higher *I*_G_/*I*_D_ ratio in rGO suggests an increase in the number of defects (*n*_D_) in the graphene layer, which led to a decrease in the distance between defects (*L*_D_), confirming the introduction of *sp* ^3^-type defects during chemical oxidation, as well as the formation of smaller *sp* ^2^ domains during the thermal reduction process.^38–41^

The Raman spectra of the HA samples display three bands associated with characteristic Raman active modes of the phosphate (PO_4_^3–^) group in the HA. The band observed at approximately 588 cm^–1^ arises from the bending (*v*_4_) mode of the O–P–O bond in the PO_4_^3–^ group. A high intense band that appears at 956 cm^–1^ is associated with a symmetrical stretching (*v*_1_) mode of the P–O bond. This band is the strongest evidence of the formation of HA.^11^ The band located at 1034 cm^–1^ is assigned to the asymmetric stretching (*v*_3_) mode of the P–O bond in the PO_4_^3–^.^42–44^ In addition, no other Raman vibrations related to other impurities or secondary phases are observed, confirming the high purity of the synthesized HA by the microwave hydrothermal-assisted coprecipitation method.^19^

The Raman spectrum of the HA/rGO composite displays characteristic bands corresponding to the Raman-active modes of each composite precursor, confirming the successful formation of the composite. Li et al.^33^ suggest that during the in situ formation of HA on rGO sheets, the oxygen-comprising functional groups on GO act as binding sites for calcium ions via electrostatic interplay. This facilitates subsequent interactions with HPO_4_^2–^ ions, leading to the formation of HA nanocrystals. Based on the morphological analysis presented in Section 3.5, the HA nanorods (∼70 nm in length) are distributed both on the surface and at the edges of the rGO sheets. This suggests an increased interaction between the oxygen-containing functional groups on rGO (particularly *sp^3^*-type defects) and HA nanocrystals compared to GO alone. This may indicate that following microwave-assisted hydrothermal reduction, only the oxygen functional groups not bound to HA are partially removed from the rGO structure, resulting in an incomplete restoration of the *sp^2^* carbon network. The *I*_D_/*I*_G_ ratio was estimated to be 1.32, indicating that the HA is bonded to the rGO. Similar results have previously been reported by other authors.^11,12,19^

### 3.3 Fourier-Transform Infrared (FTIR) Spectroscopy

Figure 4 displays the FTIR spectra of the synthesized samples. For the GO and rGO spectra, it is possible to observe the presence of peaks related to the natural vibration modes of oxygen-functional groups contained in the graphitic structure. These results confirm the successful oxidation and reduction of both GO and rGO. In the case of GO, the broad band at approximately 3300 cm^–1^ is associated with the stretching vibration of the −OH bond (hydroxyl group). At 1728 cm^–1^ is a band related to the symmetric vibration of the —C=O bond in the carbonyl and carboxyl groups.^12^ Additionally, the C–O and C–O–C stretching vibrations are displayed at 1243 and 1038 cm^–1^.^8,45^ At 1584 cm^–1^ is a band associated with the stretching vibration of the —C=C— skeleton on the graphic sheet. In the case of rGO, lower intense, and broader bands are present at 1725, 1584, and ∼1200 cm^–1^, indicating the elimination of the oxygen-functional groups from the graphitic structure by thermal reduction of GO.^8,12^

**Figure 4:**
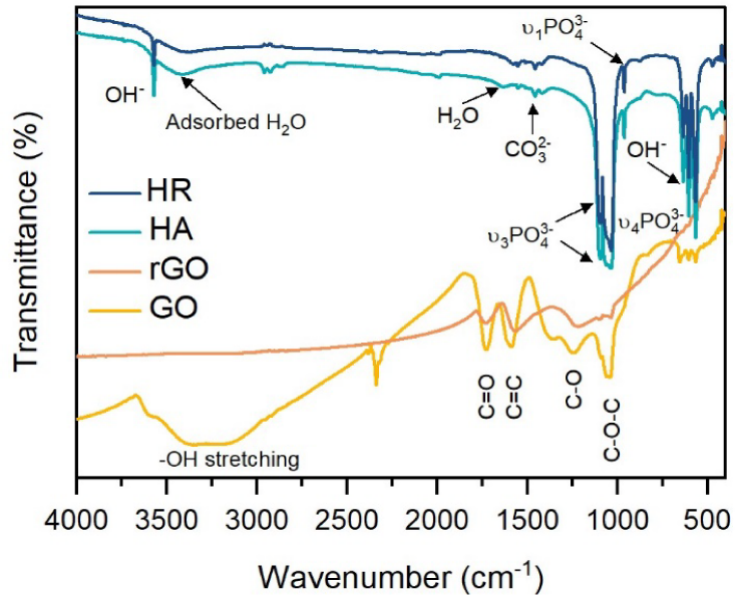
FTIR spectra of the synthesized powdered samples.

The FTIR spectrum of HA exhibits characteristic bands at 3571 and 634 cm^–1^, corresponding to the stretching and symmetric modes of hydroxyl (OH^–^) groups, confirming their presence within the HA crystal structure. A broad band at 3442 cm^–1^ is attributed to adsorbed water molecules, while the peak at 1645 cm^–1^ is related to the bending mode of water. The vibrational modes of the phosphate (PO_4_^3–^) group are observed in the spectral range between 1100 and 550 cm^–1^. Specifically, the bands at 1093 and 1037 cm^–1^ are assigned to the asymmetrical stretching (υ_3_) mode of the P–O bond, while the band at 962 cm^–1^ is assigned to the symmetrical stretching (υ_1_) mode. The asymmetrical flexion (υ_4_) mode of the O–P–O bond is found at 605 and 559 cm^–1^.^12,46,47^

The HA/rGO composite spectrum exhibits the presence of only vibrational modes of HA, which can be associated with a low concentration of rGO in the HR sample. However, according to the Raman spectra discussed above, the formation of the HA/rGO composite is achieved successfully.

### 3.4 Soft X-Ray Spectromicroscopy

Scanning transmission X-ray microspectroscopy (STXM) is a powerful technique to characterize nanomaterials since it combines the benefits of transmission X-ray microscopy with X-ray absorption spectroscopy (XAS). This technique has a high spatial resolution, which allows X-ray absorption spectra of extremely small regions (submicron) to be measured and undistorted spectra to be obtained even from samples that are too thick.^48^

Figure 5 presents the spectromicroscopic STXM results for the HA sample analyzed using principal component analysis (PCA). Figure 5a illustrates the PCA-generated cluster regions of the sample in different colors (except the background in blue), where each cluster represents a spectroscopically different sample region. Figure 5b presents the Ca L-edge and O K-edge XAS spectra obtained from the average of each cluster region in Figure 5a, and the spectra are in optical densities (O.D.), i.e., X-ray absorbance. The STXM-XAS reveals prominent peaks at the Ca L_3_ and L_2_ edges at 349.14 and 352.67 eV, respectively.^49^ Additionally, XAS of calcium phosphate (CaP) compounds such as HA typically displays two equally intense peaks, labeled a_3_ and b_3_, along with a more intense c_3_ peak, indicative of an octahedral (O_h_) crystal field geometry, as reported in previous studies.^49,50^ A corresponding set of peaks appears at the Ca L_2_ edge, although they are less distinct due to higher energy levels and reduced transition probability. In summary, all of the spectral features at the Ca L-edge, including peaks and shoulders, are observed at 346.9, 348.09, 348.35, 349.1, 351.3, 351.8, and 352.4 eV. The O K-edge XAS of the sample is dominated by pronounced peaks at 537.6 and 540.5 eV, which are due to the σ*­(P–O) bonding of the phosphate groups, confirming the presence of HA. Finally, Figure 5c presents the P K-edge XAS spectra obtained from the cluster regions in Figure 5a. The peak at 2158 eV indicates the presence of oxidized phosphorus species (P^5+^) associated with the PO_4_^3–^ groups in HA, in agreement with previous reports.^51,52^

**Figure 5:**
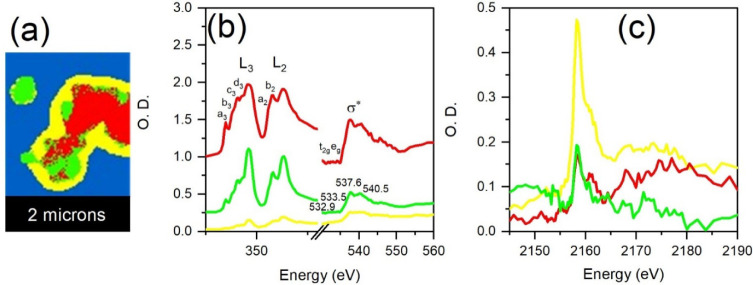
(a) STXM PCA cluster image of HA and STXM spectra of the (b) Ca L-edge and the O K-edge, as well as the (c) P K-edge of the HA.

Figure 6 presents the STXM results of the HA/rGO composite using PCA analysis. Similarly, Figure 6a illustrates the PCA-generated cluster regions of the HA/rGO sample. Figure 6b presents the C K-edge, Ca L-edge, and O K-edge XAS spectra obtained from the clusters in Figure 6a. For the C K-edge, the spectral features (280–290 eV) are attributed to transitions from the C 1s level to the π* and σ* levels of the aromatic (∼285 eV), carbonyl (∼288 eV), carboxylic (∼288.5 eV), and epoxy groups (∼289 eV) present in the rGO.^53,54^ The pronounced C 1s feature around 289 eV indicates a significant amount of carboxylic and epoxy groups in the rGO. Also, the interaction between rGO and HA is reflected in this feature due to the interaction between rGO oxygen-containing groups and the HA components, presumably Ca^2+^ species. In Figure 6b, the Ca L-edge and the O K-edge show similar spectral features to those of Figure 5b, confirming the presence of HA in the composite material. Also, Figure 6c presents P K-edge XAS features similar to those of Figure 5b, except the energy shift in the main peak, which was due to the beamline energy scale drifting during the measurement, a common issue at high energies for plane grating monochromator (PGM)-based soft X-ray beamlines. All in all, as there is no significant spectral change in the Ca L-edge and the O and P K-edges in Figure 6, compared with Figure 5, all these suggest that the HA and rGO materials interact through weak forces rather than strong covalent bonds, thus confirming the formation of a composite material.

**Figure 6:**
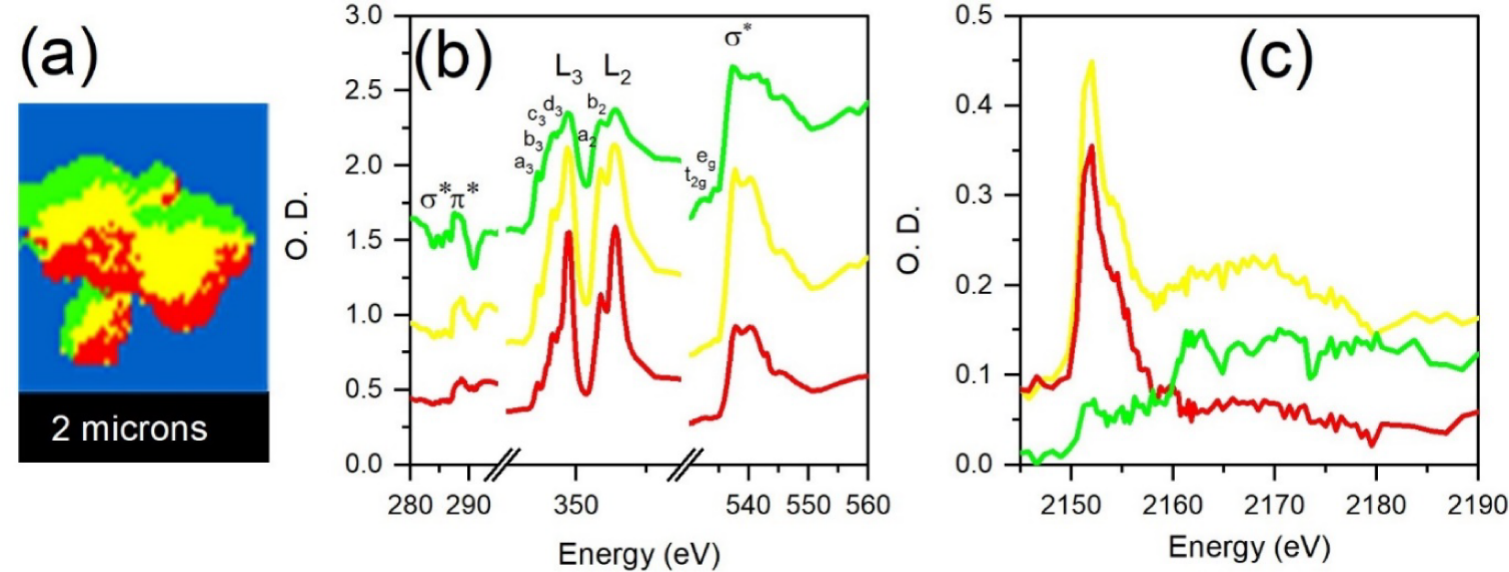
(a) STXM PCA cluster image and STXM spectra of the (b) C K-edge, Ca L-edge, and O K-edge, as well as the (c) P K-edge of the HA/rGO composite.

### 3.5 Scanning Electron Microscopy (SEM)

Field-emission scanning electron microscopy (FESEM) was utilized to investigate the morphological characteristics of the HA/rGO composite and its precursors. The obtained images are shown in Figure 7. Figure 7a,b corresponds to the GO and its reduced form rGO, respectively. Lamellar, folded, wrinkled, and disordered structures are observed, and the micrographs show these structures formed by several graphene layers.^55,56^ EDS analysis yielded C/O ratio values of 1.7 and 4.42 for GO and rGO, respectively. These values indicate that a partial removal of oxygen functional groups is performed in the case of rGO.^6,8^

**Figure 7:**
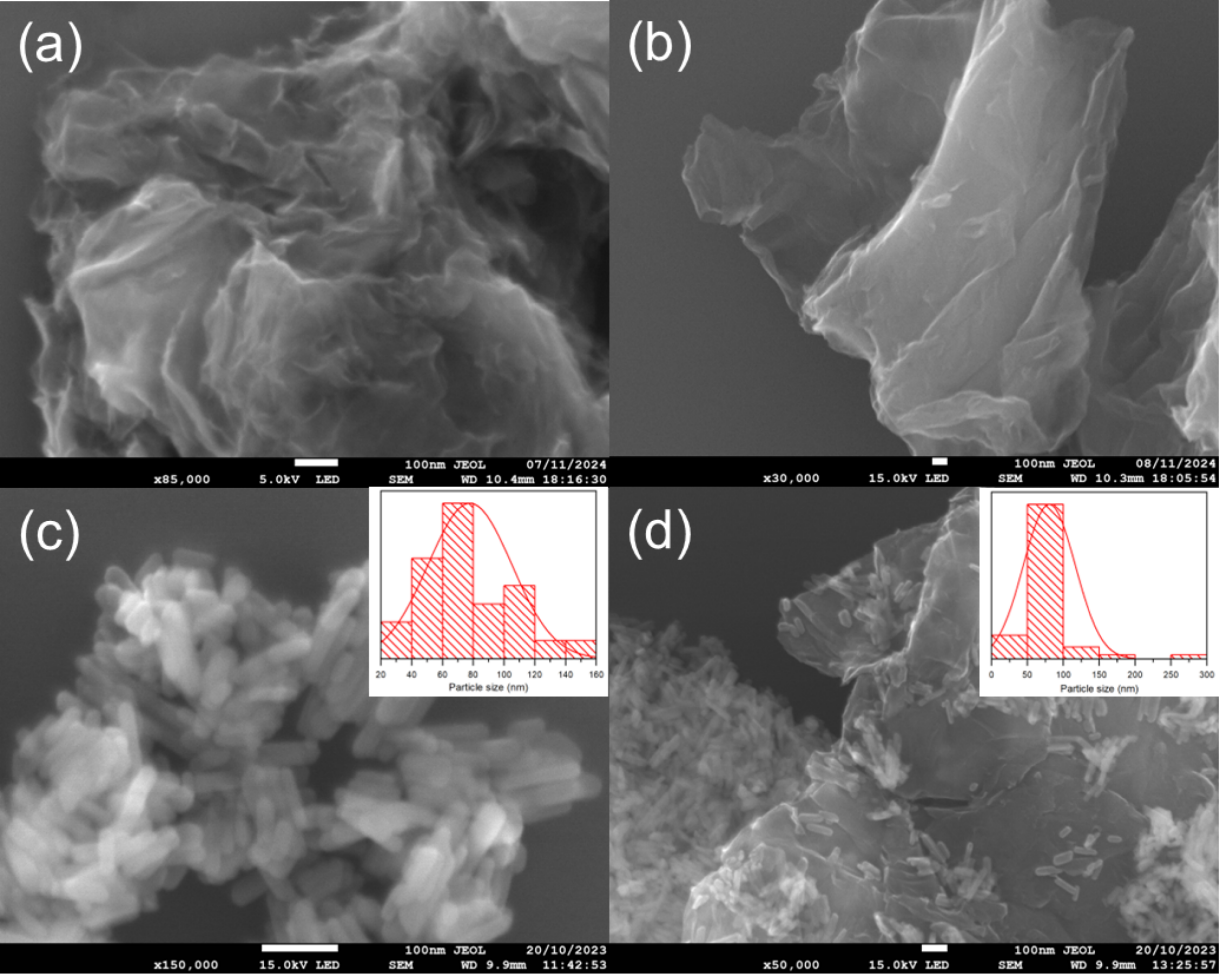
SEM images of the (a) GO, (b) rGO, (c) HA, and (d) HA/rGO composite.

Figure 7c shows the micrograph of the HA sample, where HA is observed to be forming aggregates consisting of nanorod-type nanoparticles with an average length of 70 ± 2 nm.^18^ On the other hand, Figure 7d shows the composite with HA nanorods distributed at and on the edges of the graphitic sheet of the rGO. The size of these nanorods present in the composite is estimated to be 75 ± 2 nm. Moreover, the elemental composition analysis revealed a Ca/P ratio of 1.53 and 1.55 for pure HA and the HA/rGO composite, respectively. According to McConnell,^57^ a low Ca/P ratio for hydroxyapatite is attributed to the annealing temperature. For further investigation on elemental composition analysis, the EDS spectrum and compositional analysis (atomic %) of each sample are provided in Supporting Information.

## 4 Conclusions

A high-quality HA/rGO composite material was obtained by implementing a novel microwave hydrothermal-assisted coprecipitation method, which demonstrated significant structural, optical, and morphological enhancements. X-ray diffraction analysis confirmed the hexagonal crystalline phase of HA in the composite, with an average crystallite size of 28.1 nm, and Raman spectroscopy and STXM spectra revealed vibrational and electronic transitions characteristic of HA and rGO, respectively, proving the successful incorporation of both compounds. The evolution of GO to rGO was found by FTIR analysis, which confirmed the removal of oxygen-functional groups and formation of the HA/rGO composite. SEM imaging further demonstrated the uniform distribution of HA nanorods along the surface and edges of the graphitic layers, with an average length of 75 ± 2 nm.

The improved electron transfer capabilities of the composite position it as a promising candidate for electrochemical biosensing applications, particularly in medical diagnostics and biomolecular detection. Furthermore, the synthesis approach used in this study provides a scalable and environmentally friendly route for fabricating HA-based composite materials. Future research must be conducted to investigate the electrochemical performance of HA/rGO composites in real biosensing environments, focusing on selectivity, sensitivity, and stability. Additional functionalization strategies, such as the incorporation of conductive dopants or surface modifications, may further enhance the applicability of the material in various biomedical and technological fields.

## Supplementary Material



## Data Availability

The data presented in this study are openly available in Ruíz, José (2025), “Structural and optical characterization of HA/rGO composite”, Mendeley Data, V1, doi: 10.17632/v4g673zycb.1

## Notes

The steps for the structural analysis by Rietveld refinement, the estimation of microstrain and density dislocation, a description of how soft X-ray spectromicroscopy measurements were performed for HA and HR samples, and the EDS spectra and elemental composition (atomic %) of all synthesized powdered samples (PDF)
