# Supplementary material for: In Situ Synthesis of a Hydroxyapatite and Reduced Graphene Oxide Composite for Potential Electrochemical Biosensing Applications
Source: ACS Omega. 2025 Jul 4;10(27):29680–9. doi: 10.1021/acsomega.5c03514 (PMC12268423; doi:10.1021/acsomega.5c03514)
Supplement: Supplementary file 1 [file ao5c03514_si_001.pdf]

Supporting information

In Situ Synthesis of a Hydroxyapatite and Reduced Graphene Oxide Composite for Potential Electrochemical Biosensing Applications

José J. Ruíz-Osorio[1], R. Aguilar-Sánchez[2], Rutilo Silva-González[3], Ana K. Sánchez-Hernández[1], Mohammad N. Banis[4], Jian Wang[5], M. J. Robles-Águila [1,*].

[1]Centro de Investigación en Dispositivos Semiconductores, Benemérita Universidad Autónoma de Puebla, Instituto de Ciencias, Edificio IC 6, Boulevard 14 Sur y Av. San Claudio, Col. San Manuel Puebla, C. P. 72570, México

[2]Facultad de Ciencias Químicas, Benemérita Universidad Autónoma de Puebla. Puebla, 72420, México

[3]Instituto de Física, Benemérita Universidad Autónoma de Puebla, Apartado Postal J-48, Puebla, 72570, México.

[4]Department of Mechanical and Materials Engineering, University of Western Ontario, London N6A 3K7, Canada

[5]Canadian Light Source Inc., Saskatoon, Saskatchewan, S7N 2 V3, Canada

*Email: *josefina.robles@correo.buap.mx*

Rietveld Refinement Analysis

Rietveld refinement data analysis was performed using BGMN/Autoquan software version 4.2.22 with the graphical user interface Profex version 5.2.3. The incoming crystallographic data such as cell parameters, atomic positions, and space groups were collected from Crystallography Open Database file 00-900-1233 and fixed for all refinements. The background was modeled with a 9-coefficient polynomial approach and a Pseudo-Voigt function on the profile. The XRD data refinement was performed from WMIN= 8.75 to WMAX= 67.5. Anisotropic crystallite size and isotropic microstrains were considered for line broadening, B1=0_0^0.015, k1=0_0^1, and k2=0_0^0.0001 were set. For the usual factors for the analysis of weight content, GEWICHT=SPHARP4 was corrected where we defined the harmonic spheres in order 4 as a preferred orientation correction.

Microstrain and Density Dislocation

The influence of reduced Graphene Oxide (rGO) on the Hydroxyapatite (HA) nanostructure was investigated through the estimation of lattice strain and dislocation density values from XRD results, using the Williamson - Hall (W - H) method[1] and the simplified Williamson - Smallman (W - S) approximation[2], respectively. Overlapping peak positions were obtained by Gaussian deconvolution using Origin software. The analysis was centered on the primary HA diffraction peaks at 32°, 32.4°, and 33.12° (2θ), corresponding to (3-21), (2-12) and (300) planes for HA and HR samples (Figure 1). After deconvolution of these peaks, the average Full Width at Half Maximum (FWHM) was extracted for each reflection. These values were then used to calculate the microstrain (ε) and dislocation density (δ), with the results summarized in Table S1 and Table S2.

The HR sample exhibited a reduced crystallite size alongside increase an microstrain and dislocation density, suggesting significant lattice distortion. The observed peak broadening (FWHM) likely arises from microstrain induced by the

incorporation of rGO nanosheets into the HA matrix. Specifically, the lamellar structure of rGO may introduce tensile stresses within HA's hexagonal lattice due to mismatched coefficients of thermal expansion (CTE) and interfacial strain localization.

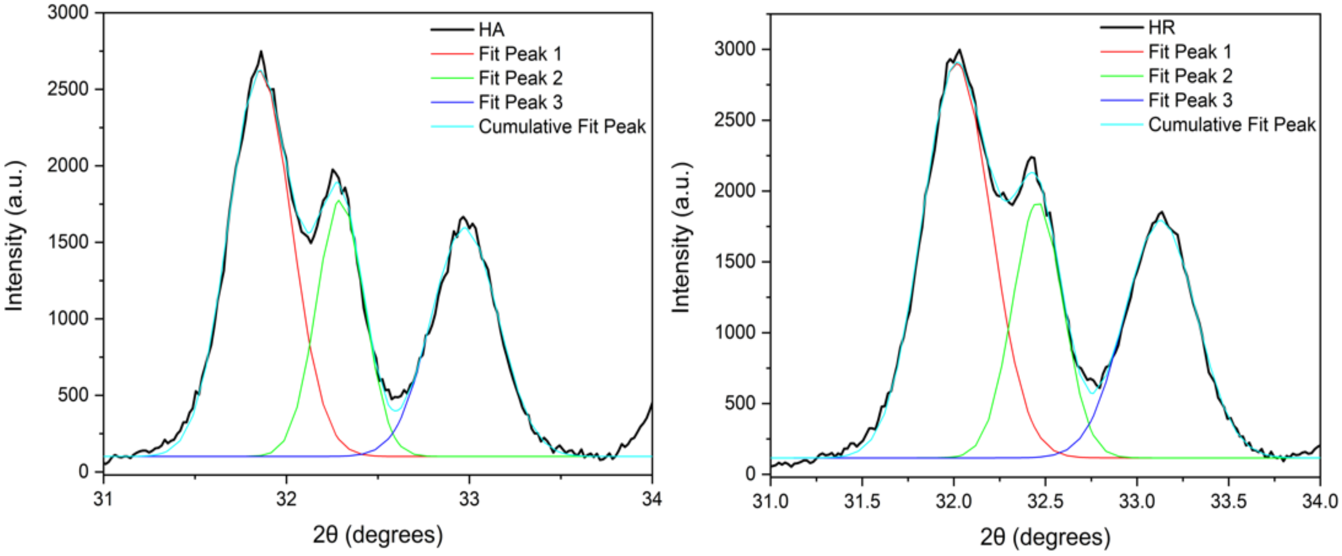

Figure S1. Deconvolution of the main XRD peaks for HA and HA/rGO composite in the range of 31 to 33.5° 2θ (°).

Table S1. Calculated Crystallographic Parameters of HA Sample

Peak	2θ position (°)	FWHM	Microstrain ($\varepsilon \times 10^{-3}$)	Dislocation density δ ($nm^{-2} \times 10^{-3}$)
**1**	31.857	0.404	0.503	1.189
**2**	32.288	0.285	0.360	2.295
**3**	32.975	0.397	0.512	1.360
	**Average**		0.458	1.615

Table S2. Calculated Crystallographic Parameters of HR Sample				
**Peak**	**2θ position (°)**	**FWHM**	**Microstrain ($\varepsilon \times 10^{-3}$)**	**Dislocation density δ ($nm^{-2} \times 10^{-3}$)**
**1**	32.007160	0.424	0.531	2.634
**2**	32.446920	0.333	0.423	1.620
**3**	33.142730	0.418	0.543	2.546
	**Average**		0.498	2.266

Scanning Transmission X-ray Microscopy (STXM)

STXM measurements of all samples for C, O, P and Ca edges were performed using the STXM end station in the 10ID-1 beamline of X-ray spectromicroscopy (SM) at the Canadian Light Source (CLS) located at the University of Saskatchewan in Saskatoon, S.K., Canada.

Data Collection and Analysis

The data set (stack) for *C, O* and *P K-edge,* and *Ca L-edge* spectromicroscopy were collected in transmission mode under the He atmosphere of the ultra-thin section used for C *K*-edge NEXAFS. Similar to C, the image sequences were recorded in two dimensions and subsequently, the monochromator was varied in energy increments from 0.15 to 0.80 eV (depending on the item analyzed).

For the hydroxyapatite (HA) sample, Ca L-edge, *O K-edge, and P K-edge* stacks were employed, as well as for the HA/rGO composite sample, the C K-edge corresponding to reduced graphene oxide (rGO) is also incorporated. First, performed on a complete micro-aggregate using a zone plate with a 500 µm focused spatial resolution, and the latest high resolution (2 µm) scans were recorded in the same micro and nano regions identified using the coordinates and central ranges (x, y) of the Ca NEXAFS. The energy flow increases the parameters and energy range (stack) images. High-resolution scans performed in CLS are provided as follows:

- ($C1s+Ca2p+O1s$) 280.0 – 560.0 eV in steps of 0.15 to 0.8 with a slit size of 20/20 μm and residence time of 1 ms, Medium Energy Grating Mode (MEG).
- ($Ps1$) 2145.0 – 2190.0 eV in steps of 0.25 with a slit size of 20/20 μm and residence time of 1 ms, High energy Grating mode (HEG).

Data analysis was performed using the aXis2000 program and a widget of Interactive Data Language (IDL) for image processing and data analysis in a Stack_Analize compatible format; PCA_GUI 1.1.1 for data analysis NEXAFS.

Energy Dispersive Spectroscopy (EDS)

The C/O and Ca/P ratios presented in the manuscript were obtained by calculating the average of three measurements taken during the compositional analysis. Figure S2 corresponds to one of the three measurements performed for elemental composition (atomic %) analysis.

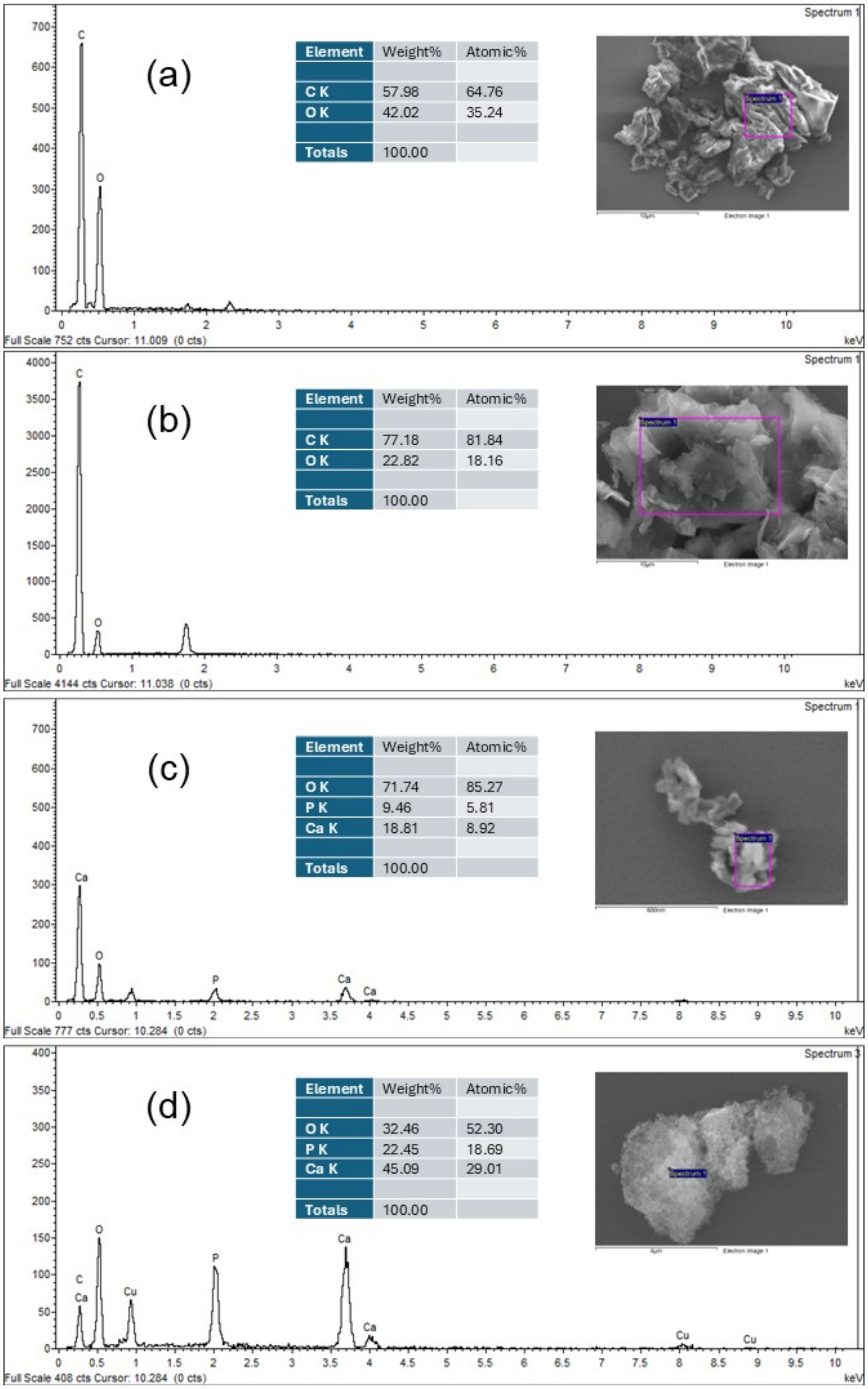

Figure S2. EDS spectra and compositional (atomic %) analysis of (a) GO, (b) rGO, (c) HA and (d) HA/rGO composite.

Availability of Data and Material: The data collected and the results from Rietveld analysis and STXM characterization are openly available in Ruíz, José (2025), “Structural and optical characterization of HA/rGO composite”, Mendeley Data, V1, doi: 10.17632/v4g673zycb.1.

References

(1) Mote, V. D.; Purushotham, Y.; Dole, B. N. *Williamson-Hall Analysis in Estimation of Lattice Strain in Nanometer-Sized ZnO Particles*; 2012. http://www.jtaphys.com/content/2251-7235/6/1/6.

(2) Williamson, G. K.; Smallman, R. E. III. Dislocation Densities in Some Annealed and Cold-Worked Metals from Measurements on the X-Ray Debye-Scherrer Spectrum. *Philosophical Magazine* **1956**, *1* (1), 34–46. https://doi.org/10.1080/14786435608238074.
